# Targeting the B-Cell Pathway in Lupus Nephritis: Current Evidence and Future Perspectives

**DOI:** 10.1155/2013/745239

**Published:** 2013-09-26

**Authors:** Panagiotis Pateinakis, Athina Pyrpasopoulou

**Affiliations:** ^1^Department of Nephrology, Papageorgiou Hospital, 56403 Thessaloniki, Greece; ^2^2nd Propedeutic Department of Internal Medicine, Hippokration General Hospital, 54642 Thessaloniki, Greece

## Abstract

Nephritis represents a frequent, severe complication of systemic lupus erythematosus. Autoantibodies appear to be fundamental in the pathogenesis of lupus nephritis. Several hypotheses are currently experimentally tested to further elucidate the direct induction of inflammation through interaction of the pathological autoantibodies with intrinsic glomerular components and the triggering of a complement-driven autoinflammatory cascade. B-cells have, in the last decade, emerged as a promising new therapeutic target, as biological treatments successfully attempting B-cell depletion, inhibition of B-cell proliferation and differentiation, or modulation of B-cell function have become bioengineered. Clinical trials have so far proved controversial regarding the efficacy of these new agents. Thus, despite the short and long-term side effects associated with immunosuppressive treatment alternative emerging treatments are still regarded “rescue” regimens in refractory patients. In an effort to accurately evaluate the potential of these therapies in lupus nephritis, several issues have been raised mainly in terms of patient selection criteria and trial duration. This review aims to expand on the proposed pathophysiologic mechanisms implicating the B-cell pathway in the pathogenesis of lupus nephritis and summarize current knowledge obtained from clinical trials introducing these biologics in its treatment. Finally, it will elaborate on potential applications of currently available biologic agents and forthcoming treatment options.

## 1. Introduction

### 1.1. Prevalence and Treatment Considerations for Lupus Nephritis

Lupus nephritis is a severe organ manifestation of systemic lupus erythematosus (SLE) that can develop in up to 70% of SLE affected individuals, with an annual incidence of roughly 0.49 per 100000 individuals per year [1, 2]. The majority of patients affected are female, younger than 50 years of age [3], due to the increased incidence of lupus among the female gender and its age distribution. However, among male patients, it is recorded as a much more frequent complication of the disease [4] and is associated with worse clinical outcome [5]. Depending on the severity of the disease, which, in turn, may be predicted from renal histology in association with other clinical and laboratory findings, 15–30% will never reach remission and will progress to end-stage renal failure [6]. 

Until the past decade, treatment was practically limited to corticosteroids, high-dose alkylating agents, and azathioprine [7]. Despite the reduction in mortality from 70% in the 1950s–1960s to <10% in recent years with the evolution of combined regimens, side-effects such as ovarian failure, infection, and bladder toxicity particularly associated with cyclophosphamide use remain considerable concerns. The introduction of mycophenolate mofetil has shown comparable efficacy in inducing remission and maintaining it, while reducing treatment-related toxicity [8]. However, high dose steroids are still administered as a necessary adjunct treatment in all induction regimens employed, with the well known short and mostly long-term side effects associated with their use [9]. Even though the precise impact of steroids in these combination treatments is unclear, data from clinical trials is currently insufficient to support earlier withdrawal [10] and/or dose reduction.

Clearly, in order to develop more targeted and less toxic therapeutic approaches, we need a better understanding of the pathogenesis of lupus nephritis [11].

## 2. Pathophysiology of Lupus (Nephritis)

### 2.1. Autoimmunity Is the Driving Force

The cornerstone of lupus pathogenesis is the compromise of self-tolerance and the production of autoantibodies [12]. Autoantibodies have been detected in affected organs from SLE patients since as early as 1967 [13], skin and kidney being the best studied organs in terms of histology of autoimmune inflammation. The presence of autoantibodies per se does not preclude development of disease [14]. “Accidentally” detected autoantibodies in otherwise normal individuals usually are of the IgM type, and react weakly with their innate antigens. Apparently, manifestation of SLE is associated with the presence of “pathogenic” autoantibodies, which interact in a high-affinity manner with their native recognition molecules. When allocated on the surface of B-lymphocytes displaying the pathological antibodies, the antigen-autoantibody interaction is augmented, and results in the stimulation of the carrier lymphocytes, their prevalence, and the initiation of an autoimmune inflammatory cascade.

Among the panel of autoantibodies characterized so far, and the clinical heterogeneity of SLE patients, antibodies reacting with DNA molecules have been invariably associated not only with the disease itself; they have also been related to its severity and organ manifestations [15]. Highly immunogenic nuclear material becomes available when the phagocytic clearance of apoptotic cells is compromised [16, 17]; impaired clearance of apoptotic nuclear material can both stimulate inflammation and drive autoantibody production [18]. Anti-DNA antibodies can form immune complexes, precipitate, and stimulate cytokine production by plasmacytoid dendritic cells, thus impacting disease, as well as becoming deposited on tissue, thus inciting inflammation and renal injury [19]. Moreover, anti-DNA antibodies may exert their immunogenic properties in a more interaction-specific manner, either through binding to cell surface antigens or to extracellular (glomerular) components directly (cross-reactivity) or indirectly (via chromatin material) [20]. After binding to cellular components, anti-DNA antibodies can be internalized, resulting in the induction of an auto-inflammatory cascade and the modulation of cell proliferation and function. Mesangial cells cultured in vitro with the addition of anti-dsDNA showed increased extracellular matrix accumulation and higher alpha-smooth muscle actin expression in the mesangial region [21]. Moreover, in mice models of immune-complex glomerulonephritis, anti-dsDNA positive animals exhibit higher alpha-smooth muscle actin expression in the mesangial region compared to anti-dsDNA negative ones.

### 2.2. Adaptive Immunity and Lupus; the B-Cell Paradigm

The breakdown of B-cell tolerance is a defining and early event in the initiation of the disease. The simplistic view that B-cell involvement in lupus development is restricted to autoantibody production has long been revisited. Indeed, lupus prone mice, genetically engineered to fail IgM immunoglobulin secretion, develop dermatitis and nephritis [22]. Several B-cell related parameters appear to become affected, including activation thresholds, B-cell longevity, and apoptotic cell processing [23, 24]. 

Among predisposing factors for the manifestation of the disease, impaired clearance of apoptotic material, such as in the case of complement (C1q) deficiencies in humans appears to be of major significance [25]. The accumulation of apoptotic debris provides large amounts of self-antigens and immune complexes, which act in a stimulatory way to various cell types, including B-cells.

In the course of autoimmune pathogenesis, activation of B-cells against self-antigens involves both impairment of antigen-driven negative selectory effects and upregulation of B-cell signaling, through loss of inhibitory Fc receptor functioning and upregulation of immature B-cell activation signaling molecules [26, 27]. The association of the detection of specific B-cell signaling gene variants with the predisposition for lupus development further confirms the pathology of B-cell signaling and activation pathways in this disorder [28].

The autoantigen activated B-cells appear to survive longer in patients with lupus compared to controls, through a pathway regulated by modified B-cell activating factor (BAFF), thus perpetuating the disease. BAFF appears to be directly involved in the selection of autoreactive clones, in switching the class of autoreactive B cells from IgM to more pathogenic IgG and, finally, in the reactivation of autoreactive class switched memory B cells and their differentiation into long-lifespan plasma cells [29]. The resulting autoinflammatory process is further amplified through disturbances in B-cell/T-cell costimulation or other immune cell activation [22]. The final end-organ damage (eg nephritis) is mediated by complement receptors, cytokines, and chemokines. 

### 2.3. B-Cell Targeted Therapies in Lupus Nephritis: Current Evidence

As expected, every factor/interaction identified as a key regulator in the initiation and perpetuation of the auto-inflammatory process was nominated as a potential therapeutic target in lupus and lupus nephritis. In the era of biologics, and in the course of a disease hallmarked by impaired adaptive immunity, several B-cell modulating treatments were tested for safety and efficacy, both in terms of controlling systemic disease activity, and of impeding organ-specific damage. 

Rituximab, a monoclonal antibody efficiently depleting CD20+ B-cells, and its fully humanized analogue, ocrelizumab, were tested in patients with lupus nephritis or extrarenal disease in three studies (LUNAR, EXPLORER, and BELONG) respectively. Patients included in these studies had moderate to severe disease. The efficient depletion of the CD20+ B cells in the significant majority of the patients was not accompanied by analogues encouraging clinical results in any of the studies [30–32]. However, statistically significant improvements in serum complement C3, C4, and antidouble-stranded DNA (anti-dsDNA) levels were observed in the rituximab treated group. The corresponding study with the fully humanized ocrelizumab was terminated early despite the numerically (but not statistically) superior response rates in the ocrelizumab versus the placebo group, due to an imbalance in serious infections in the study drug treatment group [33, 34]. Interestingly, open label studies with rituximab in lupus nephritis, in contrast to controlled trials, have shown promising results in patients with refractory disease [35], suggesting that the ideal patient pattern, which the particular therapy should address, remains to be defined.

In a recent study analyzing B-cell activating factor expression in biopsies of lupus nephritis patients and categorizing patients according to the levels of expression, biochemical markers of renal function impairment were found to be significantly associated with the increased expression of BAFF and B-cell infiltration of the biopsy specimens [36]. Interestingly, duration of lupus or nephritis, disease activity markers, as well as levels of C3/C4, did not differ between the two groups. To investigate the meaning of this finding in association of the effect of BAFF inhibition in clinical praxis, two phase III trials (BLISS-52 and BLISS-76) were conducted in autoantibody-positive lupus patients.

In both trials, belimumab treatment (BAFF-inhibitor) improved overall SLE disease activity in most common musculoskeletal and mucocutaneous organ domains. Less worsening was observed in the haematological, immunological, and renal domains [37]. However, a limitation of the post hoc analysis of the results of these two studies was the small sample size of some organ system manifestations and in terms of the effect on renal involvement, the exclusion of patients with severe lupus nephritis from the studies. The observation that BAFF levels increase in B cell depleted patients after repopulation in comparison to baseline levels, and that the increase in BAFF levels appears to significantly associate with disease flares indicates a significant involvement of BAFF in diseases activity and lupus flares [38].

CD22 is a B-cell-specific transmembrane sialoglycoprotein that inhibits the B-cell receptor complex, causing early apoptosis and thus shortening the cell's life span. In vivo antagonism of CD22 inhibits BCR-induced cell death, and prolongs the survival of B-cells in peripheral organs [39]. Epratuzumab, a bioengineered IgG1 monoclonal antibody directed against the CD22 molecule, was recently tested in several clinical trials (EMBLEM, ALLEVIA) on lupus patients with moderate to severe nonrenal disease in combination with standard treatment. Mainly through the blockade of B cell signaling and not through B cell depletion per se (or rather through preferential reduction of CD27- cells), epratuzumab clinical application appeared to be associated with dose-dependent improvement in disease activity indices and increase in adverse reactions [40, 41]. However, data is still not solid, and the issue of its effect on lupus nephritis has not been addressed yet. 

Ongoing genetic and epigenetic manipulations in mice, aiming to further unravel the B-cell related pathogenesis of lupus, will undoubtedly provide us with even more new therapeutic accessories to include in the arsenal against lupus.

## 3. Conclusions and Future Perspectives

B-cell pathology is a founding causality in the pathogenesis of lupus. Biologics specifically targeting the pathways involved in this pathophysiological condition are continuously becoming developed. Observational data from the direct application of these agents in clinical practice and controlled trials is encouraging but is not solid yet and, on occasion, raises points of awareness. 

Current research on the treatment of lupus nephritis addresses two clinically important questions: (i) whether there might be, as real life data point out, a potential indication for B-cell depleting strategies in refractory lupus nephritis, or in aggressive new onset lupus nephritis, characterized by specific and reproducible clinical, serological, and/or histopathological criteria, which could predict low responsiveness to standard treatment, and (ii) whether the addition of B-cell targeted biologics will aid in the reduction or withdrawal of steroids with sparing of all their well recognized associated side effects.

Several open label studies have indeed been able to show a beneficial effect of rituximab in patients with resistant lupus nephritis, that is, disease not responding to standard treatments [42–45]. These results, in combination with data regarding the beneficial long-term effect of rituximab on biochemical and histological renal indices [45], even in the absence of renal function improvement, support the use of rituximab in otherwise refractory lupus nephritis, at least until the scientific community can rely on a more promising agent.

Since the development of immunosuppressive agents for the treatment of nephritis and other complications of lupus and in particular in the context of transplantation, one of the major research quests has been the ability to formulate steroid-sparing regimens. The groundbreaking RITUXILUP trial introduced a steroid sparing treatment regimen in patients with active ISN/RPS class III/IV or V lupus nephritis not requiring dialysis, consisting of two doses of intravenous rituximab and methylprednisolone followed by oral mycophenolate. Early results on the first 50 patients with a median follow-up of 37 weeks demonstrate that oral steroids could safely be avoided in the treatment of active lupus nephritis [46]. 

As can be easily comprehended, the beneficial effect of disentangling lupus pathophysiology will in the future be translated in the development of more disease-specific and mechanistic-targeted treatments and the sparing of patients from the burden of agents, still considered to be indispensable for treating lupus nephritis.
